# Effect of microecological preparation supplementation on woman with polycystic ovary syndrome: A meta-analysis protocol

**DOI:** 10.1097/MD.0000000000013040

**Published:** 2018-11-02

**Authors:** Haibo Zhang, Wenting Wen, Junlong Shen, Luxia Wei

**Affiliations:** Nanjing University of Chinese Medicine, Nanjing, China.

**Keywords:** polycystic ovary syndrome, prebiotics, probiotics, synbiotics

## Abstract

**Background:**

Polycystic ovary syndrome (PCOS) is the most common endocrine disorder among women of reproductive age. PCOS not only affects female fertility, but is also associated with a variety of metabolic diseases, such as type 2 diabetes. Microecological preparations include probiotics, prebiotics, and synbiotics, and a number of studies have shown its advantages in reducing cardiovascular and cerebrovascular diseases in patients with PCOS, however, no meta-analysis has been performed to confirm that. Herein, we describe the protocol of a proposed study based on the Preferred Reporting Items for Systematic Reviews and Meta-Analyses guidelines that aims to systematically evaluate the efficacy and safety of microecological preparation supplementation in woman with PCOS.

**Methods:**

Two researchers will search 9 electronic databases (PubMed, Cochrane Library, Embase, ClinicalTrails, China National Knowledge Infrastructure, Sino Med, ScienceDirect, VIP, and Wanfang Data databases) to identify all studies that meet the inclusion criteria and were published before November 1, 2018. After information extraction and methodological quality evaluation, we will use RevMan software (version 5.3) to synthesize the data. The primary outcomes will be fasting blood glucose, total cholesterol (TC), triglycerides (TG), high-density lipoprotein cholesterol (HDL-c), low-density lipoprotein cholesterol (LDL-c), and very low-density lipoprotein cholesterol (VLDL-c).

**Results:**

This study will provide a high-quality synthesis of existing evidence on the effect and safety of microecological preparation supplementation on reducing cardiovascular risk of woman with PCOS.

**Conclusion:**

This study will determine if microecological preparation supplementation is an effective and safe intervention on reducing cardiovascular risk of woman with PCOS.

**Registration:**

PROSPERO (registration number: CRD42018108403).

## 1 Introduction

Although there are discrepancies in the reports of many earlier studies regarding the prevalence of polycystic ovary syndrome (PCOS), it is still undoubtedly the most common endocrine disorder among women of reproductive age.^[1]^ PCOS is characterized by ovulatory dysfunction and/or androgen excess or polycystic ovaries and has been associated with functional derangements of adipose tissue, metabolic syndrome, type 2 diabetes mellitus (T2DM), and an increased risk of cardiovascular disease.^[2]^

Microecological preparations include probiotics, prebiotics, and synbiotics. There has recently been growing interest in microecological preparation supplementation in patients with metabolic or endocrine diseases such as T2DM and PCOS. Studies based on gnotobiotic models and fecal microbiota transplants have demonstrated that perturbations of bacterial communities play a key role in the pathophysiology of obesity and insulin resistance.^[3]^ Bacteria produce amino acids that can be used as precursors for fatty acid synthesis, leading to the development of obesity ^[4]^ and producing symptoms of intense functional hyperandrogenism and insulin resistance in obese women.^[5]^ There are several published studies on microecological preparation supplementation in women with PCOS investigating the effects of microecological preparation supplementation on fasting blood sugar (FBS), blood lipids, and metabolism. The results of these studies need to be collectively evaluated. In our study, a meta-analysis of randomized controlled trials (RCTs) will be conducted to determine the effect and safety of microecological preparation supplementation on FBS and blood lipids in women with PCOS.

## 2 Methods

### 2.1 Inclusion criteria for study selection

#### 2.1.1 Types of studies

All randomized controlled trials (RCTs) evaluating the use of microecological preparation supplementation in the treatment of patients with PCOS will be included without language limitations.

#### 2.1.2 Types of participants

Standard diagnosis of PCOS patients will be included in the analysis, regardless of their age, ethnicity, and background, according to the National Institutes of Health,^[6]^ the European Society of Human Reproduction and Embryology/American Society for Reproductive Medicine,^[7]^ and/or the Androgen Excess Societ.^[8]^

#### 2.1.3 Types of interventions

In this study, the intervention received by patients with IBS in the experimental group will be microecological preparation supplementation, while the control group will be administered a placebo.

#### 2.1.4 Outcomes

The main endpoints will be fasting blood glucose, total cholesterol (TC), Triglycerides (TG), high-density lipoprotein cholesterol (HDL-c), low-density lipoprotein cholesterol (LDL-c), and very low-density lipoprotein cholesterol (VLDL-c), while the secondary endpoints will involve fasting serum insulin (FSI), homeostasis model assessment-insulin resistance (HOMA-IR), quantitative insulin sensitivity check index (QUICKI), body mass index (BMI), testosterone (T), and C-reactive protein (CRP).

### 2.2 Search strategy

#### 2.2.1 Electronic searches

Nine databases namely PubMed, Cochrane Library, Embase, ClinicalTrails, China National Knowledge Infrastructure, Sino Med, ScienceDirect, VIP, and Wanfang Data databases will be electronically searched will be searched from their inception to November 1, 2018. No language restrictions will be applied to the search. Two reviewers (HZ and WW) will independently search the studies. Any differences will be resolved through discussion with a third author (JS).

Search strategy of PubMed was as follows:

(1) (([probiotics] or prebiotics) or synbiotics) or microecological preparation supplementation,
(2) (PCOS) or polycystic ovary syndrome,
(3) Step 1 and step 2.

#### 2.2.2 Searching other resources

Potential eligible studies will be searched for relevant conference proceedings and reference lists of previously published reviews.

### 2.3 Data collection and analysis

#### 2.3.1 Study selection

Two researchers (HZ and WW) will work together to complete the literature search. The same 2 researchers will screen the retrieved literature by reading the titles and abstracts. The full text of relevant literature will then be read, and the studies will be selected in accordance with the inclusion criteria.

#### 2.3.2 Data extraction

Two reviewers (HZ and WW) will use a data extraction form to extract data on participants, randomization, interventions, outcomes, duration, follow-up, reasons for discontinuation, number of treatment-related adverse events, author information, and conflicts of interest. If needed, primary authors of the trials will be contacted via email to provide any missing data. Another reviewer will double check the extracted data. In cases of disagreement, the 2 researchers will crosscheck and discuss the discrepancy, or seek advice from a third party (JS).

#### 2.3.3 Processing missing data

When experimental data are missing or inadequate, we will attempt to contact the original author of the study by e-mail or telephone to obtain sufficient and comprehensive data. Incomplete data will be discarded if sufficient data cannot be retrieved.

#### 2.3.4 Risk of bias assessment

To assess the methodological quality of the included studies, 2 reviewers (HZ and WW) will independently use the Cochrane risk of bias tool to examine 7 aspects: random sequence generation, allocation concealment, blinding of participants and personnel, blinding of outcome assessment, incomplete data assessment, selective outcome reporting, and other sources of bias.

#### 2.3.5 Heterogeneity assessment and statistical analysis

Continuous data from each of the selected studies will be combined for meta-analysis using the inverse variance method. Results will be expressed as mean difference (MD) and 95% confidence intervals (CI). Study-to-study variation will be assessed using the chi-square statistic. The hypothesis tested will be that the studies are all drawn from the same population—that is, from a population with the same effect size. A two-tailed *P* < .05 will be required to statistically significant. A fixed effects model will be used when there is no statistically significant heterogeneity between individual study results; otherwise a random effects model will be applied. All results will be combined for meta-analysis with RevMan software (version 5.3).

#### 2.3.6 Subgroup analysis

If significant heterogeneity is observed in the included studies, subgroup analysis will be performed based on age, interventions, controls, and outcome measurements.

#### 2.3.7 Assessment of reporting biases

If there are a sufficient number of articles (>10) included under the same endpoint addressing the same question, a funnel plot will be used to measure publication bias.

#### 2.3.8 Sensitivity analysis

If the primary outcome analyses involve a large number of included studies and a large degree of heterogeneity, the included studies will be investigated individually to increase the stability of the final results.

#### 2.3.9 Grading of quality of evidence

The Grading of Recommendations Assessment, Development, and Evaluation will be utilized for assessing the quality of evidence for the main outcomes. The quality of evidence will be categorized as high, moderate, low, or very low.

#### 2.3.10 Ethics and dissemination

All data included in this study are derived from published literature and do not include patient personal data, so no ethical approval is required. The final meta-analysis results will be published in a peer-reviewed journal.

## 3 Discussion

PCOS is a heterogeneous and chronic condition; it can be divided into 3 broad segments: reproductive manifestations, metabolic features, and psychological sequelae. The spectrum of clinical features varies across the life cycle, in adolescence PCOS exhibits reproductive and psychological manifestations, which over time transition to frank infertility and metabolic complications, however, in obese adolescent metabolic implications of PCOS such as impaired glucose tolerance (IGT), diabetes mellitus (DM), and metabolic syndrome can be present.^[9]^

The recent studies has revealed that microecological preparation supplementation had its advantages in reducing cardiovascular and cerebrovascular diseases in patients with PCOS, but there are differences between the results of different studies. Therefore, we plan to conduct a systematic review and meta-analysis of existing clinical studies to objectively evaluate the effect and safety of microecological preparation supplementation in reducing cardiovascular risk of woman with PCOS. However, the proposed study has certain limitations; the compositions of microecological preparation supplementation might be vary greatly, there is no guarantee that same drug dosage will have been used on the patients, and individual differences in populations of different ethnicities may affect the final study results.

## Author contributions

**Conceptualization:** Haibo Zhang, Wenting Wen, Junlong Shen.

**Data curation:** Haibo Zhang, Wenting Wen.

**Formal analysis:** Haibo Zhang, Wenting Wen, Luxia Wei, Junlong Shen.

**Funding acquisition:** Wenting Wen, Junlong Shen, Luxia Wei.

**Fundingacquisition:** Junlong Shen, Luxia Wei, and Wenting Wen

**Investigation:** Haibo Zhang, Wenting Wen, Junlong Shen, Luxia Wei.

**Methodology:** Wenting Wen.

**Writing – original draft:** Haibo Zhang, Wenting Wen, Junlong Shen, Luxia Wei.

**Writing – review & editing:** Junlong Shen, Luxia Wei.

Haibo Zhang orcid: 0000-0003-4483-6662

Wenting Wen orcid: 0000-0002-3930-818X

Junlong Shen orcid: 0000-0001-7498-3730.
